# The Antioxidant Potential of Graviola and Its Potential Medicinal Application

**DOI:** 10.3390/nu15020402

**Published:** 2023-01-12

**Authors:** Beata Olas

**Affiliations:** Department of General Biochemistry, Faculty of Biology and Environmental Protection, University of Lodz, Pomorska 141/3, 90-236 Lodz, Poland; beata.olas@biol.uni.lodz.pl; Tel./Fax: +48-42-6354485

**Keywords:** antioxidant, graviola, oxidative stress

## Abstract

Graviola (*Annunona muricata* L.), a plant growing in tropical regions, has many names and a range of ethnomedicinal uses. The leaves are used to treat insomnia, diabetes, cystitis, and headaches, the crushed seeds have anthelmintic properties, and the fruits are used in the preparation of ice creams, candy, syrups, shakes, and other beverages. The key active components are believed to be annonaceous acetogenins, with more than 100 such compounds having been isolated from *A. muricata*. The plant is also a source of a range of phenolic compounds, essential oils, alkaloids, flavonol triglycosides, and megastigmanes, together with various minerals, including Mg, Fe, Cu, K, and Ca. Its key phenolic compounds are rutin, kaempferol, and quercetin. This paper provides an overview of the current state of knowledge about the antioxidant properties of various graviola organs and their major constituents, based on a review of various electronic databases. However, few findings have been obtained from clinical trials, and few in vitro and animal studies suggest that graviola preparations have antioxidant properties; as such, the antioxidant potential of graviola, and its safety, remain unclear.

## 1. Introduction

**Figure 1:**
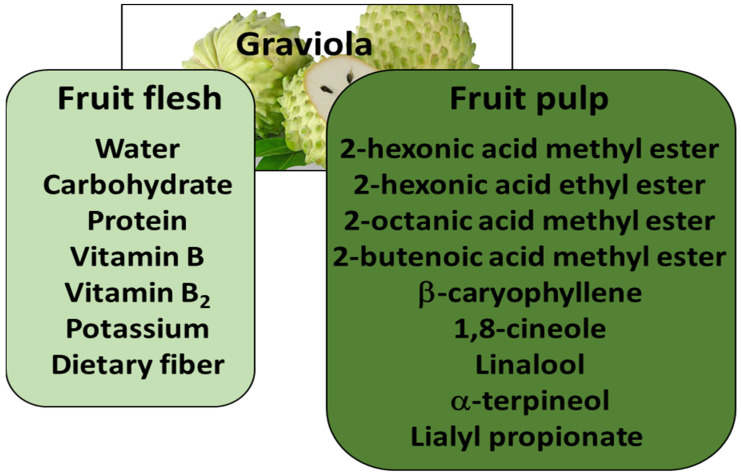
Chemical composition of graviola fruit flesh and fruit pulp (adapted from ref.: [1]).

*Annunona muricata* L. (also known as graviola, soursop, guanabana, paw-paw, and sirsak) is an evergreen member of the *Annonaceaea* that grows throughout tropical and subtropical regions in Malaysia, India, and Nigeria. The tree bears large, heart-shaped, edible green fruits, ranging from 15 to 20 cm in diameter and weighing between 0.4 and 4 kg depending on the country. Graviola fruit flesh and pulp are rich in water, carbohydrates, salts, and vitamins, and are ideal for juices and drinks. They may also be readily eaten. The chemical compositions of the fruit flesh and pulp are given in Figure 1.

The fruits are widely used in the preparation of various foods, such as ice creams, syrups, nectars, jams, jellies, candies, and beverages. Graviola is very popular in the USA, for example in the form of capsules (stem and leaf powder), and tea under various trade names (for example, Soursop leaf tea). In addition, all parts of the plant, but mainly the leaves, seeds, roots, and bark, are used as natural medicines for treating diarrhea, arthritic pain, fever, neuralgia, dysentery, malaria, rheumatism, parasites, and skin rashes. The ethnomedicinal uses of graviola and the biological properties of its various organs are described in more detail by Moghadamtousi et al. [2]. 

Recent years have seen considerable interest in identifying the antioxidant properties of natural products. Indeed, nature has been a source of therapeutic agents for thousands of years, and a large number of modern medicines, including supplements with antioxidant properties, were originally obtained from natural sources. Antioxidants prevent oxidative damage to the most important cellular macromolecules, such as lipids, proteins, and nucleic acids, by soaking up reactive oxygen species (ROS) produced in various biochemical processes, as well as by reducing oxygen concentrations and binding metal ions to prevent radical formation. 

Oxidative stress may be indicated by markers associated with three key types of damage to macromolecules: lipid oxidation markers such as malondialdehyde (MDA), conjugated dienes or F_2_-isoprostanes, protein modification markers indicating protein fragmentation, nitrated proteins, carbonylated proteins or thiol group oxidation, and various markers associated with nucleic acid damage [3]. These parameters not only have diagnostic value, but they may also be useful indicators of the need for antioxidant supplementation.

Therefore, in recent years, great importance has been attached to the consumption of fresh vegetables and fruits, as they are a source of naturally-occurring antioxidants. Research suggests that this increased intake of fruits and vegetables may be associated with a reduced incidence of disorders induced by ROS, including cardiovascular disorders, cancer, neurodegenerative disorders, and inflammatory processes. Indeed, the consumption of antioxidants in the diet may have a significant effect on the prophylaxis and progression of various diseases associated with oxidative stress [4,5,6]. 

Plants are known to have high levels of vitamins A, C, and E, which may act as antioxidants. However, various authors have attributed the health benefits of whole foods to their complex mixtures of phytochemicals, including phenolics, which are known to have strong antioxidant properties. Moreover, the antioxidants obtained from whole foods are believed to bestow greater beneficial effects than individual supplements [7,8]. 

Interestingly, various parts of graviola are available in pharmacies, herbal and online shops as dietary supplements in the form of products, such as juices, syrups, tinctures, and capsules. However, little is known of their exact antioxidative potential, and a review by Moghadamtousi et al. [2] examining the current state of knowledge about the antioxidant properties of graviola organs and their major constituents includes only a short chapter on this topic. This review itself consisted of a search of PubMed, ScienceDirect, Web of Knowledge, Google Scholar, and SCOPUS, based on the terms “graviola”, or “*Annona muricata*”, or “antioxidant”, or “oxidative stress”. The last search was run on 10 November 2022. 

## 2. Phytochemical Studies of Different Parts of Graviola

**Figure 2:**
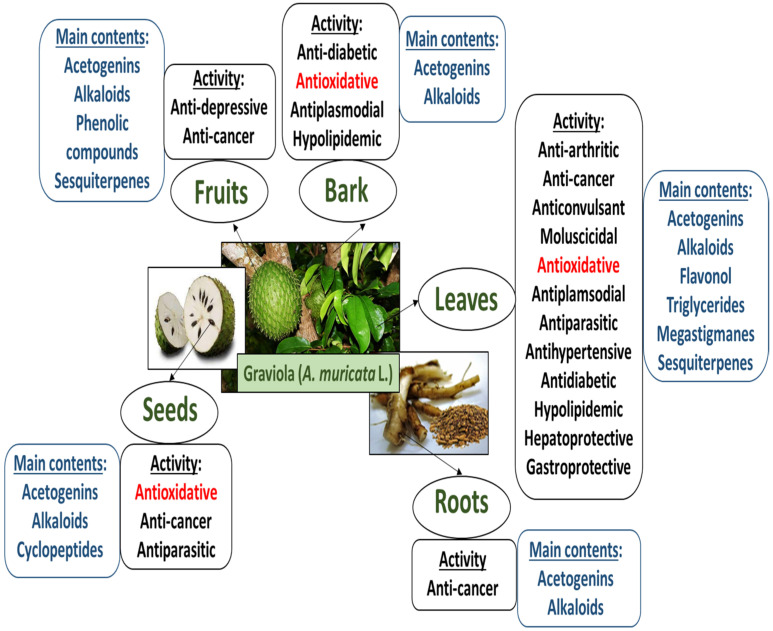
Main contents and main biological properties of various parts of graviola (adapted from ref.: Moghadamtousi et al. [2]).

Phytochemical analyses have revealed that graviola is rich in annonaceous acetogenins; however, its chemical composition varies between the organs (Figure 2). More than 100 of these compounds have been isolated from the various organs of *A. muricata* and these have been identified as the main bioactive constituents of graviola. Annonaceous acetogenins are a unique class of C-35/C37 secondary metabolites derived from long-chain (C-32/C34) fatty acids via the polyketide pathway. They are usually formed from a combination of acids; they are characterized by a 2-propanol unit at C-2, which forms a methyl-substituted α,β-unsaturated γ-lactone. However, annonaceous acetogenins are generally waxy substances and are difficult to work with because of poor water solubility [1,9].

Graviola is also a source of various substances including phenolic compounds, essential oils, alkaloids, flavonol triglycosides, and megastigmanes. It also provides various minerals such as Mg, Fe, Cu, K, and Ca.

The major phenolic compounds are rutin, kaempferol, and quercetin. These are known to have a range of antioxidant, anti-cancer, and anti-platelet activities [10,11,12].

The main biological properties of various parts of graviola are presented in Figure 2. For example, the leaves are used to treat insomnia, diabetes, cystitis, and headaches, while the crushed seeds have anthelmintic and antiparasitic properties, and the roots and bark are believed to bestow hypoglycemic, anti-inflammatory, and hypertensive effects [2,9,12,13]. Recently, Kim et al. [14] reported that the ethanol graviola leaf extract attenuates hepatic lipogenesis and adipogenesis in mice fed a high-fat diet. Aqueous graviola leaf extract (50, 100, and 150 mg/kg) also protected obese C57BL/6 mice from the effects of metabolic disorders caused by a high-fat diet [15]. A number of papers also indicate that graviola has anticancer potential [1,13,16,17,18,19].

## 3. Antioxidant Potential of Graviola

The main biological activities (including antioxidant potential) of fruits, leaves, and other parts of graviola are presented in Figure 2. The antioxidant potential of graviola preparations has been examined in various in vitro and in vivo studies; their antioxidative potential may vary depending on the method of extraction, type of preparation, and the type of cell and tissue model in which they are tested.

### 3.1. In Vitro Studies

Baskar et al. [20] examined the in vitro antioxidant potential of leaves from various *Annona* species, *viz*. *A. muricata, A. squamosal*, and *A. reticulate* using various biomarkers of oxidative stress, including hydroxyl radical level, nitric oxide (NO) level, and 2,2′-azino-bis(3-ethylbenzothiazoline-6-sulfonic acid (ABTS) level. It was found that *A. muricata* leaf extract (500 µg/mL) had the strongest antioxidant potential. 

The reducing power of the leaf extract was found to be 216.41 µg/mL gallic acid equivalents (GAE) for an aqueous extract and 470.51 µg/mL for the ethanolic extract [21]. The antioxidant activity values were 2.0456 mg/mL and 0.9077 mg/mL for the ethanolic and aqueous extracts, respectively (in vitro). In addition, the ethanolic extract was found to be selectively cytotoxic to three tumor cell lines, including the breast cancer cell lines MDA and SKBR3.

George et al. [22] report that both the aqueous and methanolic extracts from graviola leaves have antioxidant properties, and can protect DNA against H_2_O_2_ damage. HPLC analysis confirmed the presence of various phenolic compounds in the used extracts, including flavones, isoflavonols, and flavanones. In addition, a strong positive correlation was seen between the total phenolic content and the radical scavenging properties of each extract measured by hydroxyl scavenging activity assay (HRSA), DPPH radical scavenging assay, and ferric reducing antioxidant property (FRAP). Moreover, the authors note that methanolic extract offers superior protection against H_2_O_2_-induced DNA damage.

In another in vitro experiment, Son et al. [11] studied the antioxidant activity of graviola leaf extracts in HepG2 cells. They found a 50% ethanol extract to be superior to a steam extract in scavenging nitrogen and peroxyl radicals. The ethanol extract also upregulated Nrf2 and superoxide dismutase (SOD).

Other authors indicate that extracts from graviola stem bark and seeds have antioxidant potential in vitro [23,24,25,26]. Olakunle et al. [26] report that 25–100 µg/mL ethanol extract, a source of phenolic compounds from graviola bark exhibited free radical-scavenging activity in a concentration-dependent manner, as indicated by 2,2-diphenyl-1-picrylhydrazyl (DPPH) radical assay. The tested extract (200 mg/kg b.w.) also demonstrated antioxidant potential in CCl_4_ treated rats.

### 3.2. In Vivo Studies

Oxidative stress is known to play an important role in the pathogenesis of ulcerative colitis (UC) [27]. Helal et al. [28] report that graviola leaves (100 mg/kg/day; for seven days) significantly alleviated acetic acid-induced oxidative stress in a rat model of UC. The group treated with graviola demonstrated 60% and 23% reductions in colonic MDA and NO content, respectively, compared to the untreated controls. In addition, graviola treatment significantly increased colonic GSH concentration compared to controls. The total phenolic compound and total flavonoid concentrations in the tested graviola extract were 30% and 10% by weight, respectively. Helal et al. [28] propose that graviola may attenuate acetic acid-induced UC by modulating the hedgehog signaling pathway; indeed, oxidative stress-stimulated UC is known to inactivate the hedgehog signaling pathway by suppressing the nuclear expression of various target genes including B-cell lymphoma 2 (Bcl-2).

Al-Syaad et al. [29] report the effect of graviola extract on oxidative stress-associated hyperglycemia in type 1 diabetes in male rats. Diabetes was induced by a single injection of streptozotocin (40 mg/kg, i.p.), and then plant extract (100 mg/kg/day) was orally administrated for 30 days. The authors suggest that the tested extract may ameliorate the hepatic damage resulting from diabetes by the modulation of oxidative stress. For example, graviola extract significantly reduced lipid peroxidation indicated by MDA level and restored the activity of various antioxidant enzymes in the liver, including SOD, catalase (CAT), glutathione reductase (GR), and glutathione peroxidase (GPx). The extract was also found to restore the hepatic content of reduced glutathione (GSH). However, the study did not describe the chemical composition of the tested extract.

Alsenosy et al. [30] examined the effect of graviola (100 mg/kg/day; orally once daily) on testicular oxidative stress of streptozotocin-induced diabetic rats. The oxidative stress of testis tissue was evaluated by various biomarkers, including MDA, NO, and GSH levels. Four-week graviola administration significantly restored testicular GSH concentration and total SOD activity in the rats. These results may indicate the protective effect of graviola against the harmful significant enhancement in testicular antioxidant enzymes. Unfortunately, the study did not include a description of the chemical content of the graviola leaf preparation.

Other results indicate that graviola extract (200 mg/kg b.w.) administration normalizes oxidative stress parameters in hepatic injury induced by monosodium glutamate (MSG; 2.4 gm/kg b.w.) in rats [31]. MSG and graviola extract were administered orally for eight weeks. The results indicate that oral administration significantly decreases MDA concentration in rats compared to the MSG group. Graviola administration also decreased the concentrations of other biomarkers of oxidative stress, but this effect was not statistically significant. Unfortunately, again, the authors do not describe the chemical content of the graviola extract.

Zeweil et al. [32] found that graviola leaf extract (200 mg/kg body mass) attenuates 7,12-dimethylbenz[a]anthracene (DMBA)-induced breast cancer by augmenting apoptosis, the antioxidant pathway and downregulating estrogen receptors in rats. In this study, fifty female Wistar rats were allocated into four groups: (1) control group, (2) DMBA-treated group, (3) DMBA plus graviola extract (two times weekly at the age of 37 days until the end of the experiment), and (4) DMBA plus graviola extract (two times weekly at the age of 57 days until the end of the experiment). After the 30-week experimental period, blood samples were collected. The authors observed that the graviola extract ameliorates the DMBA effects on the activity of antioxidant enzymes, for example, CAT, SOD, and glutathione S-transferase. This extract also decreased lipid peroxidation.

As the anti-diabetic mechanisms of graviola leaf extract are believed to be associated with their influence on lipid metabolism and hepatic glucose, Son et al. [12] also investigated the protective effect of the extract on hepatic damage in diabetic mice. The extract contained rutin, kaempferol-3-*O*-rutinoside, quercetin, muricoreacin, kaempferol, annonacin, and annonacinone identified by HPLC; the total phenolic compound content was 80.1 mg GAE/g. Diabetes was induced by the combination of a high-fat diet with a double streptozotocin injection (60 mg/kg BW) in C57BL/6 male mice. The animals were then administered graviola extract (50 or 100 mg/kg BW, daily) for nine weeks. It was found that the used extract, especially at the lowest dose (50 mg/kg BW), reduces oxidative stress in hepatic tissue, as measured by 4-hydroxynonenal (4-HNE) and protein carbonyl levels. The antioxidant properties of the used extract appear to be associated with the reduction of other oxidative stress-related factors, including nuclear respiratory factor 2 (Nrf2) and NAD(P)H quinone oxidoreductase 1 (NQO1).

The antioxidant potential of different graviola preparations in various in vivo models is summarized in Table 1.

## 4. Conclusions

This review presents new data regarding the biological properties of graviola preparations. These preparations are believed to have health benefits, which have been attributed to a range of biological activities, such as their antioxidant properties; these have been largely attributed to their phenolic compound content. However, so far, graviola preparations have only exhibited antioxidant potential in in vitro and animal models; no clinical studies have been performed on human subjects with diseases associated with oxidative stress, and the safety and efficacy of the preparations remain undetermined. However, a systematic review by Chan et al. [9] indicates that graviola leaf extract was safe in humans at an oral dose of 540 mg/day when taken for up to 30 days. However, our findings concerning the antioxidant activity of graviola require further confirmation in future clinical trials aimed at identifying the possible beneficial effects of graviola. 

## Figures and Tables

**Table 1:** The antioxidant potential of graviola preparation in in vivo models.

Graviola Preparation	Dose	Days	Subjects	Effects	References
Graviola leaf extract capsules^®^ from Bio Nutrition Company (Island Park, NY, USA) (total phenolic compounds—30%, and total flavonoids—10%)	100 mg/kg/day	7	Rats with ulcerative colitis (n = 28)	Decrease in colonic MDA and NO content; increase in colonic GSH	[28]
Graviola extract (chemical content: undefined)	100 mg/kg/day	30	Rats with type 1 diabetes (n = 12)	Decrease in MDA concentration and increase in antioxidant enzymes and GSH in the liver	[29]
Graviola dry extract^®^ (product code: 912943735; Origini Naturali Company (Quarrata, Pistosia, Italy)) (chemical content: undefined)	100 mg/kg/day	28	Diabetic rats (n = 10)	Increase in testicular GSH and total superoxide dismutase activity	[30]
Graviola dry extract^®^ (product code: 912943735; Origini Naturali Company (Quarrata, Pistosia, Italy)) (chemical content: undefined)	200 mg/kg/day	28	Rats with hepatic injury (n = 6)	Decrease in MDA level in hepatic tissue	[31]
Extract from graviola leaves (total phenolic compounds: 80.1 gallic acid equivalent/g)	50 or 100 mg/kg/day	72	Type 2 diabetic mice (n = 68)	Decrease in 4-HNE and protein carbonyls in hepatic tissue	[12]
Ethanol extract of graviola stem bark (chemical content: undefined)	200 mg/kg b.w.	45	Rats treated with CCl_4_	Inhibition of plasma lipid peroxidation (measured by MDA)	[26]
Extract of graviola leaves (chemical content: undefined)	200 mg/kg b.w.	50	Rats treated with DMBA-induced breast cancer	Inhibition of lipid peroxidation and increase in activity of antioxidant enzymes	[32]
